# Exploring the Relationship between the Inhibition Selectivity and the Apoptosis of Roscovitine-Treated Cancer Cells

**DOI:** 10.1155/2013/389390

**Published:** 2013-04-04

**Authors:** Chunying Cui, Yaonan Wang, Yuji Wang, Ming Zhao, Shiqi Peng

**Affiliations:** ^1^College of Pharmaceutical Sciences, Capital Medical University, Beijing 100069, China; ^2^Medical Experiment and Test Center, Capital Medical University, Beijing 100069, China

## Abstract

The antitumor activity of roscovitine was tested in four cervical carcinoma cells: C33A, HCE-1, HeLa, and SiHa. The effects of roscovitine on ATP Lite assay, cell cycle, and apoptosis were assessed. The Sub-G_1_ DNA content occurred great increasing, and this indicates that apoptosis was induced quickly in HeLa cells, but slowly in the other cells. The morphological observation results showed that roscovitine induced apoptosis and cell death in the cervical carcinoma cells. Results revealed that roscovitine exhibited selective cytotoxicity towards 4 cervical carcinoma cells, and the cells showed different morphologic and apoptotic changes at the same concentration. It was estimated that cervical carcinoma cells responded differently to roscovitine because of differences in apoptotic and genetic background in different cervical carcinoma cells. This study suggested that roscovitine had the potential to be a chemotherapeutic agent against cervical carcinoma.

## 1. Introduction

Cyclin-dependent kinase inhibitors have the potential to induce cell cycle arrest and apoptosis in cancer cells [1]. Roscovitine, a potent and selective inhibitor of Cdk2 and Cdc2, has demonstrated selective inhibition of Cdk enzymes over related kinases. It has been reported that roscovitine does cause not only cell cycle arrest, but also apoptosis in cancer cells [2, 3]. In in vitro study, it has been shown that roscovitine has cytotoxic activity against a lot of human tumor cells, as well as in tumor xenograft models [4, 5]. Roscovitine is currently undergoing phase II clinical trials as a treatment for nonsmall cell lung cancer and nasopharyngeal cancer [6, 7]. 

In this study, we investigated whether roscovitine could inhibit the tumor growth and exhibit cytotoxicity in cervical carcinoma cell lines: C33A, HCE-1, HeLa, and SiHa. In addition, we are interested in elucidating the biochemistry of apoptosis of roscovitine on these cell lines. Our data showed that roscovitine can inhibit tumor cell proliferation in dose- and time-dependent manner in cervical carcinoma cells. Roscovitine can induce cell cycle arrest and apoptosis in 4 cervical cells but showed selective sensitivity. We estimated that cervical carcinoma cells responded differently to roscovitine because of differences in apoptotic and genetic background. These results also suggest that roscovitine may be a selective and effective chemotherapeutic agent against cervical carcinoma. 

## 2. Materials and Methods

Roscovitine was purchased from Sigma-Aldrich (CAS: 186692466, USA). C33A, HCE-1, HeLa, and SiHa cell lines were purchased from the Institute of Basic Medical Sciences Chinese Academy of Medical Sciences and Shanghai Sanqiang Analysis Company. Dulbecco's modified Eagle medium (DMEM), fetal bovine serum (FBS), and trypsin were purchased from Hyclone Laboratories Inc. (USA). Penicillin and streptomycin were purchased from Sigma Chemical Company (USA). Dimethyl sulfoxide (DMSO) was purchased from AppliChem GmbH Company (Germany). 3-(4,5-Dimethylthiazol-2-yl)-2,5-diphenyl-tetrazolium bromide (MTT) and acridine orange were purchased from Amresco company (USA). Protease inhibitor cocktail (1%, Cat No: 539134) was purchased from Merck (USA). All reagents were of chemical grade unless otherwise specified.

### 2.1. Cell Culture and Reagents

A 10 mmol/L roscovitine stock solution of the compound was prepared in DMSO and diluted to a different concentration. The final concentration of DMSO in culture medium was ≤0.3%. Four cervical carcinoma cells (C33A, HCE-1, HeLa, and SiHa) were maintained in RPMI-1640 media containing 10% fetal bovine serum, 2 mmol/L L-glutamine, 100 U/mL penicillin, and 100 *μ*g/mL streptomycin. Cells were cultured in an incubator at 37°C under 5% CO_2_ in air.

### 2.2. Assessment of Cell Viability (Dose and Time Relationship)

 C33A, HCE-1, HeLa, and SiHa cells (5 × 10^4^/well) were grown in 24-well plates and treated with roscovitine (0–30 *μ*mol/L) or DMSO (0.3%, final concentration) for 48 and 72 h. Attached cells were released by trypsin and combined with nonadherent cells. After centrifugation, cells were resuspended in PBS and treated with 0.2% trypan blue. Experiments were performed in five replicates independently. For cell growth inhibition experiment, cells were seeded in 24-well culture plates at a density of 5 × 10^4^/well. After 0, 2, 4, 6, 12, 24, and 72 h, 20 *μ*mol/L of roscovitine was added into the wells. Cell number and cell viability were determined using haemocytometer and trypan blue dye exclusion test. 

### 2.3. MTT Assay

 C33A, HCE-1, HeLa, and SiHa cells were seeded into 96-well plates and incubated overnight at 37°C. Roscovitine was added to cells (in 5 replicates) and incubated for 72 h at 37°C. 3-[4,5-Dimethylthiazol-2-yl]-2,5-diphenyltetrazolium bromide (MTT) was made up as a stock of 2 mg/mL in cell media and filter sterilized. Media were removed from cells, and MTT solution was then added at 50 *μ*L per well and incubated in the dark at 37°C for 4 h. MTT solution was removed, and MTT dye was solubilised with 50 *μ*L/well of DMSO with agitation. Measure the absorbance at 562 nm and then determine the IC_50_ (concentration of roscovitine which inhibits cell growth by 50%).

### 2.4. Flow Cytometry

Apoptotic effects of roscovitine on cervical carcinoma cells were detected by flow cytometry. Cervical carcinoma cells in logarithmic phase of 5 × 10^5^/mL were incubated into 6-well cell culture plate and were cultured at 37°C in an incubator with 5% CO_2_ for 24 h. With the culture medium removed, the volume of 2 mL containing 20 *μ*mol/L roscovitine was added into each well. The cells were cultured at 37°C in an incubator with 5% CO_2_ for 0, 6, 12, 24, 48, and 72 h, respectively, collected, respectively, and then centrifuged at 2000 g for 5 min. With the supernatant removed, the cell concentration was diluted to 1 × 10^6^/mL. The media binding reagent with 10 *μ*L of AnnexinV-FITC was added. The cells were incubated in darkness for 15 min and centrifuged at 3000 g for 5 min, and then the supernatant was removed. The cells were resuspended in the 0.5 mL binding buffer. The cell suspension was added with 10 *μ*L PI on ice. The cells were protected from light and detected by flow cytometry.

### 2.5. ATP Lite Assay

ATP is a sign of cell viability, and it exists in all living cells with metabolism. As cells undergo necrosis or apoptosis, the ATP concentration decreases rapidly. The Luciferase and D-luciferin contained in the ATP Lite kit react with ATP to produce fluorescence. The reaction formula is as follows:

(1) ATP+D-Luciferin+O2→Oxyluciferin+AMP+PPi+CO2+Light

Fluorescence intensity was measured by PerckinElmer 2030 Multilabel Reader. 100 *μ*L cells suspension were seeded at 3 × 10^5^/mL in 96-well culture plate. After 24 h, the cells were added with 100 *μ*L culture medium containing roscovitine 10, 20, 30, 40, 50, and 60 *μ*mol/L, respectively. The blank group was added with media, and the control group was added with 0.5% DMSO. Each group had 5 repeats. The cells of C33A and HeLa were cultured for 48 h. Temperatures of ATP Lite buffer, cell lysis solution, and lyophilized substrate solution were balanced to room temperature (RT). ATP Lite buffer with the volume of 5 mL was added into the lyophilized substrate solution. Cell lysis solution with the volume of 50 *μ*L was added into each well. The mixture was vibrated at 1000 g for 5 min by microvibration to dissolve the cells and to stabilize ATP. The prepared matrix solution with the volume of 50 *μ*L was added into each well. The mixture was vibrated at 1000 g for 5 min and was protected from light for 10 min to measure its fluorescence intensity.

### 2.6. Morphological Observation

The concentration of C33A and HeLa cells in logarithmic phase was diluted to around 5 × 10^4^/mL. 2 mL of the C33A and HeLa cell suspension was placed into 6-well culture plate and covered by the cover slips. The cells were cultured at 37°C in an incubator with 5% CO_2_ for 24 h. Then the culture medium was removed. 20 *μ*mol/L roscovitine was added into each well, respectively, with the group of 0.3% DMSO as the control group. Two groups were cultured for 0 and 24 h, respectively. The cover slip with growing cells was selected, washed with PBS for 3 times to remove serum, fixed with 95% ethanol for 15 min, treated by 1% acetic acid for 30 s, then stained by 2 × 10^−4^ mol/L acridine orange for 30–60 s, processed by 1 mol/L CaCl_2_ for 30–120 s, and washed by PBS for 3 times. A slide dropped with 200 *μ*L glycerol was covered lightly, as the excess liquid was sucked by filter paper. The picture was taken by Confocal microscope at the excitation wavelength of 488 nm and the emission wavelength of 561 nm.

### 2.7. Statistics

 All experiments were performed at least 5 times. Data are expressed as means ± SD. A *P value of <0.05 was considered statistically significant, and **P value of <0.01 was considered very significant.

## 3. Results

### 3.1. The Effect of Roscovitine on Viability and Growth

**Figure 1:**
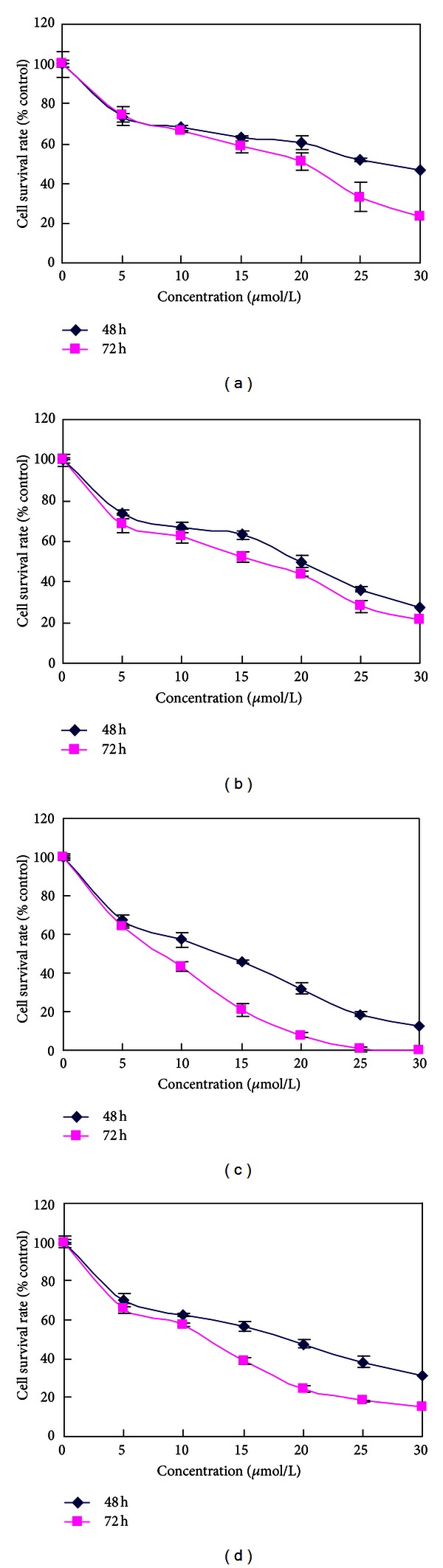
Survival rates (%) after 4 cervical carcinoma cell lines were incubated with various concentrations of roscovitine. (a), (b), (c), and (d) represent C33A, HCE-1, HeLa, and SiHa cell lines. Data are presented as the mean ± SD. (*n* = 5).

**Figure 2:**
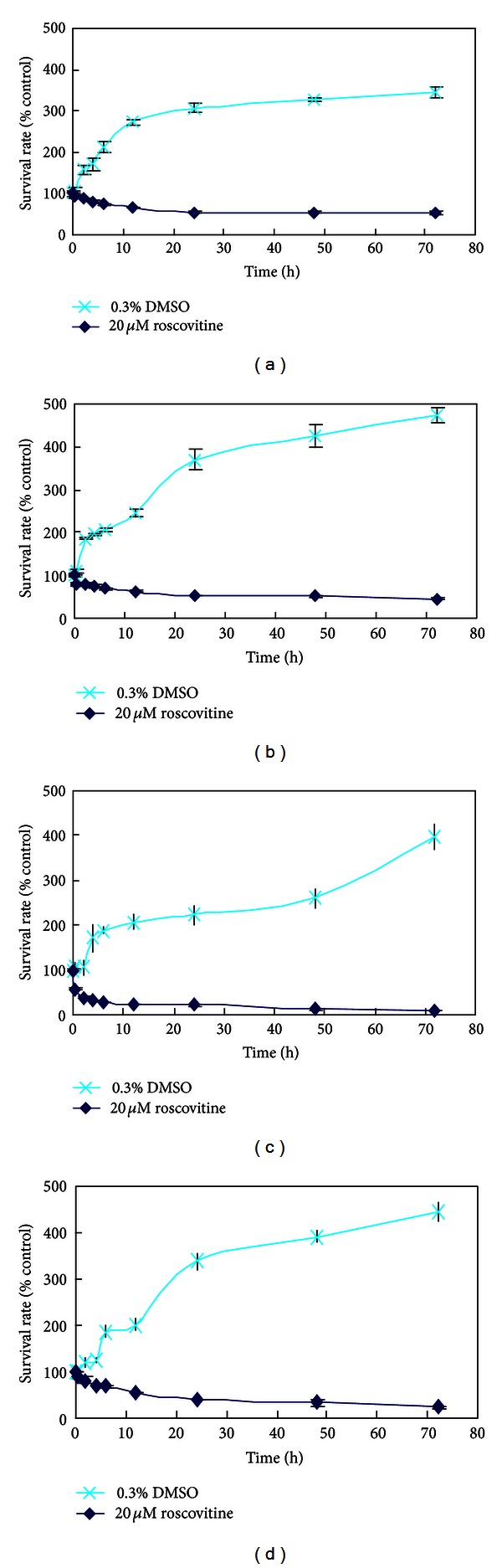
Survival rates (%) after 4 cervical carcinoma cell lines were incubated with 0.3% DMSO or 20 *μ*mol/L roscovitine at various time points. (a), (b), (c), and (d) represent C33A, HCE-1, HeLa, and SiHa cell lines. Data are presented as the mean ± SD. (*n* = 5).

The growth of cells was inhibited in dose-dependent manner after exposure to roscovitine for 48 h and 72 h ranging from 0 to 30 *μ*mol/L (Figure 1). HeLa cells were most sensitive. The antiproliferation effect of roscovitine was evaluated by measuring the growth rates and treated with 20 *μ*mol/L roscovitine. Treatment with roscovitine caused a time-dependent inhibition of cell growth in accordance with the cell viability assay during 72 h as compared with control (Figure 2). 

### 3.2. Cytotoxicity Assay

**Figure 3:**
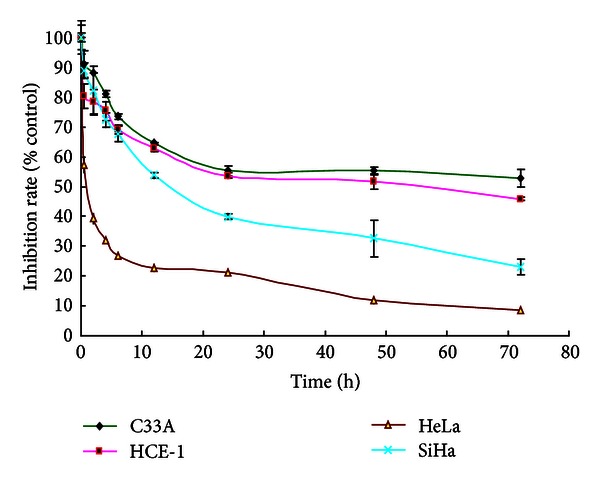
Inhibition rates (%) of cervical carcinoma cells treated with 20 *µ*mol/L roscovitine at various time points. The results are expressed as the mean ± S.D. (*n* = 5).

Cytotoxicity was determined by MTT assay following 72 h incubation with roscovitine. The IC_50_ values of C33A, HCE-1, HeLa, and SiHa cells were 22.09 ± 3.29, 21.21 ± 1.96, 13.79 ± 3.30, and 16.88 ± 7.39 *μ*mol/L, respectively, as shown in Figure 3. 

### 3.3. Cell Cycle Effects

**Figure 4:**
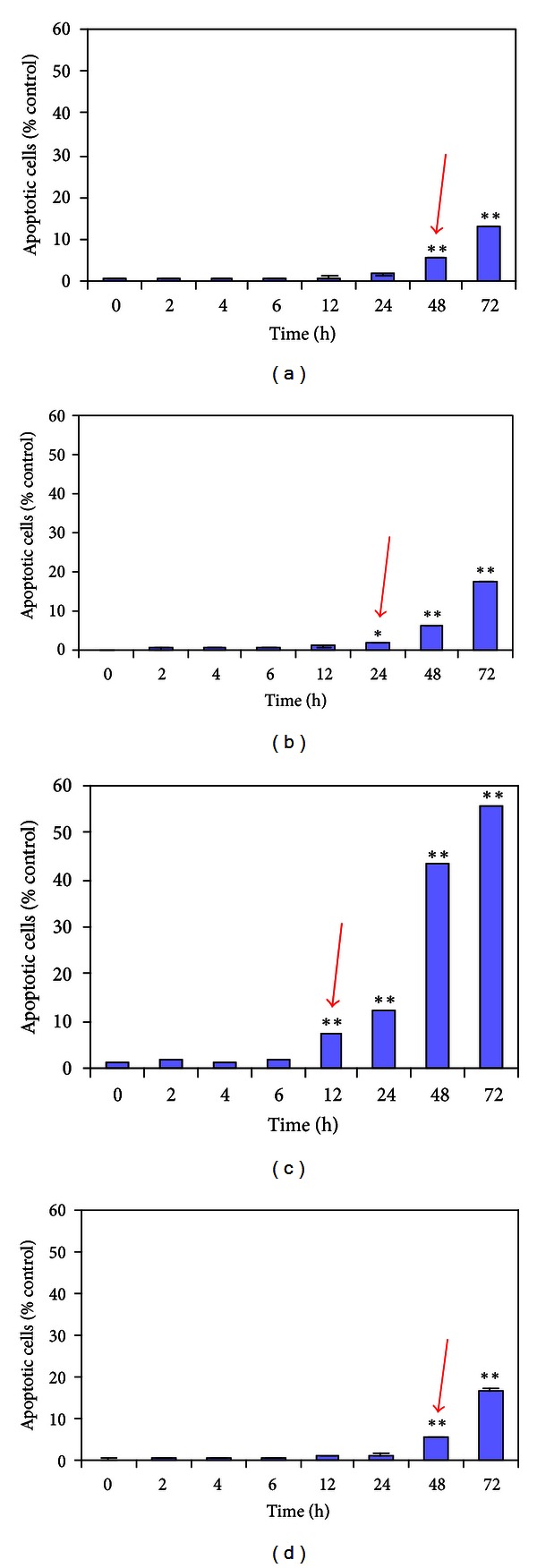
Effect of 20 *μ*mol/L of roscovitine on the apoptosis of C33A, HCE-1, HeLa, and SiHa cancer cells. Data are expressed with mean ± SD. (*n* = 5).

We analyzed the cell cycle profiles of growing cervical cell lines exposed to 20 *μ*mol/L roscovitine. In C33A, HCE-1, and SiHa cell lines, the Sub-G_1_ DNA content was remarkable and apparently increased during 24–48 h, indicating that apoptosis occurred (Figure 4). In HeLa cell lines, we found that the Sub-G_1_ DNA content occurred great increase within 12 h. The results indicated that roscovitine induced apoptosis in four cervical carcinoma cells, and HeLa is the most sensitive to roscovitine.

### 3.4. ATP Lite Assay

**Figure 5:**
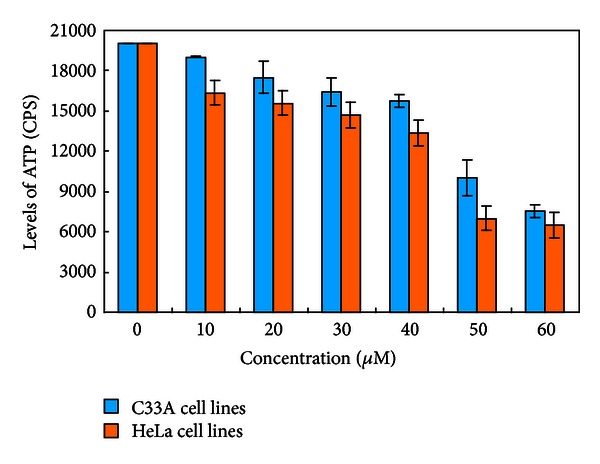
Cytotoxicity assay using ATP Lite approach: CPS changes of cervical cells treated with various concentrations of roscovitine. Data are presented as the mean ± SD (*n* = 5).

To understand the reason of the cancer cells exhibiting different sensitivity to roscovitine, the most sensitive HeLa cells and the most resistant C33A cells were used as the representatives to do the ATP Lite and morphological study. With ATP assay, it was confirmed that roscovitine significantly inhibited the growth of both C33A and HeLa cells. The intracellular ATPase activity decreased significantly as the concentration of roscovitine increased, as shown in Figure 5. The inhibition effect of roscovitine in HeLa cells is higher than that in C33A cell lines in a dose-dependent manner. 

### 3.5. Morphological Observation

**Figure 6:**
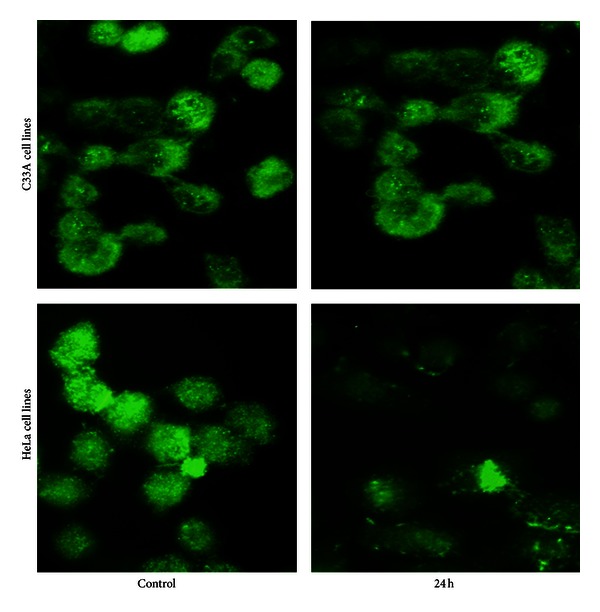
The apoptotic cell photos were taken by fluorescence microscope after C33A and HeLa cells were incubated with 20 *μ*mol/L roscovitine at 0 (control) and 24 h.

The C33A and HeLa cells stained with acridine orange fluorescence were observed by Confocal microscopy. Their nuclei presented homogeneous fluorescence of green light while in the apoptotic cells, due to the chromatin pyknosis or the broken fragments of unequal size, the apoptotic body was formed. They were stained with acridine orange and presented the deep and dense fluorescence or the granular fluorescence of green. The fluorescence in necrotic cells decreased or even disappeared. C33A and HeLa cells treated with roscovitine were taken pictures at 0, 24 h, the apoptosis feature was demonstrated clearly, as shown in Figure 6. The results indicated that the cells treated with roscovitine showed cell membrane blister, cell shrinkage, and apoptosis. Those indicated that roscovitine induced apoptosis in both two cell lines, and HeLa cells were more sensitive to roscovitine. 

## 4. Discussion

 Roscovitine, an olomoucine-related purine analogue derived from 6-DMAP and isopentenyladenine that competes with ATP for its binding site on Cdks, has been developed as a Cdk inhibitor [8, 9]. Roscovitine treatment induced not only cell cycle arrest, but also apoptosis in various type cell lines [4, 10, 11]. In this study, we showed for the first time that the novel CDK inhibitor roscovitine inhibits cervical tumor cell proliferation in dose- and time-dependent manner and induced apoptosis rapidly *in vitro* in cervical carcinoma cells by a mechanism that involved apoptosis.

 We found that roscovitine exhibited selective cytotoxicity towards cervical cells, and the cells showed different morphologic and apoptotic changes at the same concentration. This observation was confirmed by flow cytometry and indicating the apoptotic mechanism of roscovitine. Published paper using roscovitine on head and neck squamous carcinoma cells also showed two possible results [12–14]. Firstly, cells may show apoptosis concurrently with cell cycle arrest. This significance is not entirely clear but would suggest chemosensitivity. Secondly, it showed a prolonged cell cycle arrest preceding apoptosis. The results suggest that cervical carcinoma cells may respond differently to roscovitine because of the mechanism and relationship of apoptosis and cell cycle arresting. From our study, roscovitine can inhibit tumor cell proliferation in dose-dependent and time-dependent manner and exhibited difference cytotoxicity in different cervical cell lines. Cervical carcinoma cells occurred apoptosis with cell cycle arrest concurrently. As such, roscovitine will be a selective and effective chemosensitivity drug for cervical carcinoma therapy.
